# ­­Effect of multimodal cues from a predatory fish on refuge use and foraging on an amphidromous shrimp

**DOI:** 10.7717/peerj.11011

**Published:** 2021-03-12

**Authors:** Maria E. Ocasio-Torres, Todd A. Crowl, Alberto M. Sabat

**Affiliations:** 1 Department of Natural Sciences, Ana G. Mendez University, Gurabo, Puerto Rico, United States of America; 2 Southeast Environmental Research Center, Florida International University, Miami, FL, United States of America; 3 Department of Biology, Florida International University, Miami, FL, United States of America; 4 Department of Biology, Universidad de Puerto Rico, San Juan, Puerto Rico, United States of America

**Keywords:** Antipredator defenses, Chemical ecology, Inducible defenses, Kairomones, Predation risk, Predator cues, Predator recognition, Alarm cues

## Abstract

**Background:**

Prey can alter their behavior when detecting predator cues. Little is known about which sensory channel, number of channels, or the interaction among channels that shrimp species use to evaluate the threat from predators. The amphidromous shrimp *Xiphocaris elongata* has an induced defense, an elongated rostrum, where predatory fishes are present. We sought to test if kairomones or visual cues when presented singly from fish either eating flakes or shrimp, had more effect on altering the temporal feeding and refuge use patterns of long-rostrum (LR) *X. elongata*. We were also interested in elucidating potential interactions among cues when presented simultaneously in different combinations (kairomones + visual + mechanosensory, kairomones + alarm + visual, kairomones + alarm, kairomones + visual) on the same response variables. We expected that when presented alone kairomones will significantly increase refuge use and decrease foraging, particularly late at night, in comparison to visual cues alone, and that multiple cues when presented simultaneously will further increase refuge use and decrease foraging at night.

**Methods:**

We exposed shrimp to individual or multiple cues from the predatory fish mountain mullet, *Augonostomus monticola*. We examined shrimp behavior with respect to refuge use and foraging activity during four time periods (after sunset, nighttime, sunrise, and sunset) in a 24-hour period.

**Results:**

Shrimp presented fish visual and chemical cues singly did not differ from one another but differed from control shrimp (no cues) with respect to refuge use or foraging. The number of shrimp using refuge in the treatment with most cues (KVM: kairomones+ visual + mechanosensory) was higher than in all the treatments with less cues. A significant decline in foraging was observed when multiple cues were presented simultaneously. The highest number of shrimp foraged one hour after sunset and at nighttime. A significant interaction was observed between cue treatments and time periods, with shrimp in the KVM treatment foraging less and using more refuge late at night and at sunrise than shrimp in other treatments or time periods.

**Conclusions:**

The observation that fish chemical and visual cues when presented singly produced similar refuge use and foraging patterns was contrary to expectation and suggests that visual and chemical cues, when presented alone, provide redundant information to *X. elongata* with regards to predation threat. The significant increase in refuge use and reduction in foraging observed in the KVM treatment suggest multimodal signal enhancement in the perception of threat. This makes evolutionary sense in “noisy” environments, such as streams, where detection, localization, and intention of predators is much improved when cues are received through multiple sensory channels.

## Introduction

Predation is an important evolutionary and ecological force because it not only shapes the behaviors, physiologies, morphologies, and life-histories of their prey, but also impacts prey population dynamics, community composition, and ecosystem processes (Black, 1993; Pestana, Baird & Soares, 2013). Predators can have indirect effects on prey (those not resulting in prey mortality) by producing visual, mechanic or chemical cues or a combination of these cues that induce changes in prey behavior (e.g., spatial or temporal segregation, changes in activity patterns, decreased movement, increased refuge use), morphology (e.g., long spines, production of helmets, thickened shells) or life-history (e.g., reproduction or growth delay) (Havel, 1987; Ives & Dobson, 1987; Adler & Harvell, 1990; Harvell, 1990; Aiken, 1998; Wisenden, 2003; Creel & Christianson, 2008; Hein, 2009; Ocasio-Torres, Crowl & Sabat, 2014). These changes in prey phenotypes may lead to trait-mediated top-down trophic cascades because predator presence may lower prey feeding activities and indirectly reduce the flow of energy from lower trophic levels (Berger, 2010; Harvey & Menden-Deuer, 2012). Detection of cues indicating increased risk of predation is crucial for prey to enhance their survival, and to alter the potential impacts that predators have on community and ecosystem dynamics (Vos et al., 2004); van der (Stap et al., 2007; Ferrari, Wisenden & Chivers, 2010; Pestana, Baird & Soares, 2013).

Many studies on the detection of predation risk in aquatic systems have focused on how detecting chemical cues from predators (kairomones) and from injured conspecifics (alarm cues) induces behavioral, morphological or life-history changes in the potential prey (Crowl & Covich, 1994; Ferrari, Wisenden & Chivers, 2010; Parsons et al., 2017). Perhaps the most documented example of chemical detection in aquatic systems is the planktonic cladoceran *Daphnia* spp., which can change its behavior, morphology, and life history when detecting chemical cues from predatory fish and insect larvae, as well as conspecifics under predatory attack (Dodson et al., 1994; Ślusarczyk, 1999; Laforsch, Beccara & Tollrian, 2006; Tollrian & Leese, 2010). Detection of chemical cues from predators or conspecifics is highly useful: (1) in aquatic systems like rivers where brown runoff water and thick riparian cover can affect and even impede visual detection of predators and of predator–prey encounters that are upstream of potential prey (Petranka, Kats & Sih, 1987; Crowl & Covich, 1990; Dodson et al., 1994; Hickman, Stone & Mathis, 2004), (2) in situations when visual cues are unreliable indicators of predation risk from fast-moving predators (Hemmi, 2005), and (3) for prey that are most active at night and can only rely on nonvisual cues to avoid predators at that time (Bouwma & Hazlett, 2001).

Multiple signals perceived by different sensory channels can interact and generate responses that are more complex than when the signals are perceived singly (Partan & Marler, 1999; Weissburg, Smee & Ferner, 2014). For example, an aquatic prey may feel more threatened and significantly increase or enhance refuge use when smelling and seeing a predator than when only seeing or smelling it. Among the benefits of multimodal signals in the context of predator–prey interactions (reviewed by Weissburg, Smee & Ferner, 2014) are that prey are able to better detect and localize predators and reduce uncertainty, particularly in “noisy” environments. There is increasing evidence that prey can indeed use multichannel cues to their advantage (Stauffer & Semlitsch, 1993; Crowl & Covich, 1994; Hazlett, 1999; Bouwma & Hazlett, 2001; Lehtiniemi, 2005; Kim et al., 2009; Hein & Crowl, 2010). However, understanding the complex interactions that can occur among different sensory channels and the myriad of responses elicited by prey remains a challenge (Weissburg, Smee & Ferner, 2014).

The tropical amphidromous shrimp species, *Xiphocaris elongata,* exhibits two morphs that are related to the presence and absence of predatory fishes (Covich et al., 2009; Ocasio-Torres, Crowl & Sabat, 2014). These shrimp possess a short rostrum above geomorphic barriers like steep waterfalls that impede the upstream migration of native diadromous predatory fishes but have a longer rostrum below barriers where diadromous predators move freely (Covich et al., 2009). It has been experimentally demonstrated that kairomones from the predatory fish *Agonostomus monticola* affect the upstream migration of post-larval *X. elongata* (Kikkert, Crowl & Covich, 2009) and induce long rostrums in juvenile and adult *X. elongata* (Ocasio-Torres, Crowl & Sabat, 2014). Predation experiments have shown that the long rostrum in *X. elongata* is an effective antipredator defense against *A. monticola* (Ocasio-Torres et al., 2015b). Daytime foraging activities of long-rostrum (LR) adult shrimp, as well as leaf-litter processing by short-rostrum (SR) adult shrimp decrease in the presence of *A. monticola* (Ocasio-Torres, Crowl & Sabat, 2015a). Studies that incorporate nighttime (when *X. elongata* are mostly active) and daytime (when *A. monticola* are mostly active) observations to examine the effect of one predation-related cue versus multiple cues on the behavior of *X. elongata* have not been completed (Pringle et al., 1993; Johnson & Covich, 2000; Scatena & Johnson, 2001; Hein, 2009; Ocasio-Torres et al., 2015b).

Our first objective was to determine if visual or kairomones (chemical cues from predatory fish), when presented alone, elicit more refuge use and less foraging in long-rostrum (LR) *X. elongata.* Secondly, we were interested in elucidating potential interactions among predator and prey cues when presented simultaneously in different combinations (kairomones + visual + mechanosensory, kairomones + alarm + visual, kairomones + alarm, kairomones + visual) on the same response variables. The final objective was to determine if predator cues, either singly or in the above combinations, affect differently refuge use and foraging patterns of LR *X. elongata* at different time periods (after sunset, nighttime, sunrise, and sunset). To accomplish these three objectives, we ran 24-hour experiments in laboratory aquaria where we exposed shrimp to either multiple or individual cues from *A. monticola* either feeding on flakes or on *X. elongata* shrimp and measured the number of shrimp using refuge or foraging.

We predicted that (1) chemical cues from fish (hereby kairomones) will increase refuge use and decrease foraging more than visual cues from fish, (2) multiple predation cues will have an enhancement effect (sensu Partan & Marler, 1999) on refuge use, and (3) refuge use and foraging will be affected mostly at nighttime. Knowledge of how predation-related cues in aquatic systems generate a behavioral response in their prey will contribute to our general understanding of the ecological and evolutionary mechanisms responsible for predator–prey communication and specific understanding of their consequences in aquatic habitats (Crowl & Covich, 1994; Dodson et al., 1994; Ocasio-Torres, Crowl & Sabat, 2014).

## Materials and Methods

### Study organisms

*Xiphocaris elongata,* yellow-nosed shrimp, are amphidromous shrimp. They spend most of their adult life in fresh waters where the females carry their eggs until hatching (Fièvet, Dolédec & Lim, 2001). The larvae then drift downstream to the estuary before migrating upstream as post-larval juveniles (March & Pringle, 2003; Kikkert, Crowl & Covich, 2009). *Xiphocaris elongata* forage on leaf matter, fruits, flowers, algae, biofilm, particulate organic matter, and dead animals (March & Pringle, 2003; Cross et al., 2008; Ocasio-Torres, Crowl & Sabat, 2014). They are most active at night though their foraging activities also occur throughout the day (Pringle et al., 1993; Johnson & Covich, 2000; Scatena & Johnson, 2001; Hein, 2009; Ocasio-Torres, Crowl & Sabat, 2015a). Their distribution is limited to the West Indies (Fryer, 1977). *Agonostomus monticola* (Family: Mugilidae)*,* mountain mullets, are a visual, pelagic, and amphidromous fish that feed on shrimps, aquatic insects, plant material, algae, and biofilm (March & Pringle, 2003; Matamores et al., 2009; Smith & Kwak, 2014). *Agonostomus monticola* are one of the main predatory fishes in the fresh waters of Puerto Rico (Covich et al., 2009; Ocasio-Torres, Crowl & Sabat, 2015a; Ocasio-Torres et al., 2015b) and their distribution has been reported in the West Indies, countries in northern South America (e.g., Colombia, Venezuela), Central America, and southern and eastern states of the United States (e.g., Florida, North Carolina) (Aiken, 1998; Matamores et al., 2009).

### Animal collection

We collected the fish and shrimp in rivers of eastern Puerto Rico using an LR-20B electrofisher. We followed the procedure suggested by its user’s guide, which recommends starting with a frequency of 15 Hz, a 10 percent duty cycle and a voltage of 50 volts. We increased voltage, duty cycle, or frequency until taxis was detected (Smithroot, Inc, 2018). *Agonostomus monticola* were collected in riffles downstream of waterfalls in Río Sabana. LR *X. elongata* were collected in pools downstream of waterfalls in Río Espíritu Santo where predatory fishes like *A. monticola* are common (Ocasio-Torres, Crowl & Sabat, 2015a). Fish and shrimp were collected in different rivers because fish were more accessible in riffles of Sabana, while shrimp were more accessible in pools of Espíritu Santo. No genetic structuring is expected in fish or shrimp among different rivers due to high gene flow and metapopulation dynamics given their amphidromous life-history (Engman, Kwak & Fischer, 2017). LR *X. elongata* were used in our experiments given that they are the only *X. elongata* phenotype that is constantly exposed to predatory fishes in the wild. The sizes of the fish and shrimp chosen for the experiments were the most common sizes collected and are comparable to sizes reported by Cooney & Kwak (2013; e.g., fish total length: 17–23 cm) and Covich et al. (2009; e.g., shrimp post-orbital length: 8–11 mm for LR; shrimp rostrum length: 10–14 mm for LR). The animal collection methods in this research were approved by the Department of Natural and Environmental Resources of Puerto Rico with the permit numbers O-VS-PVS15-SJ-00728-10022015 and R-VS-PVS15-SJ-00516-11042016.

### Procedure

**Figure 1:**
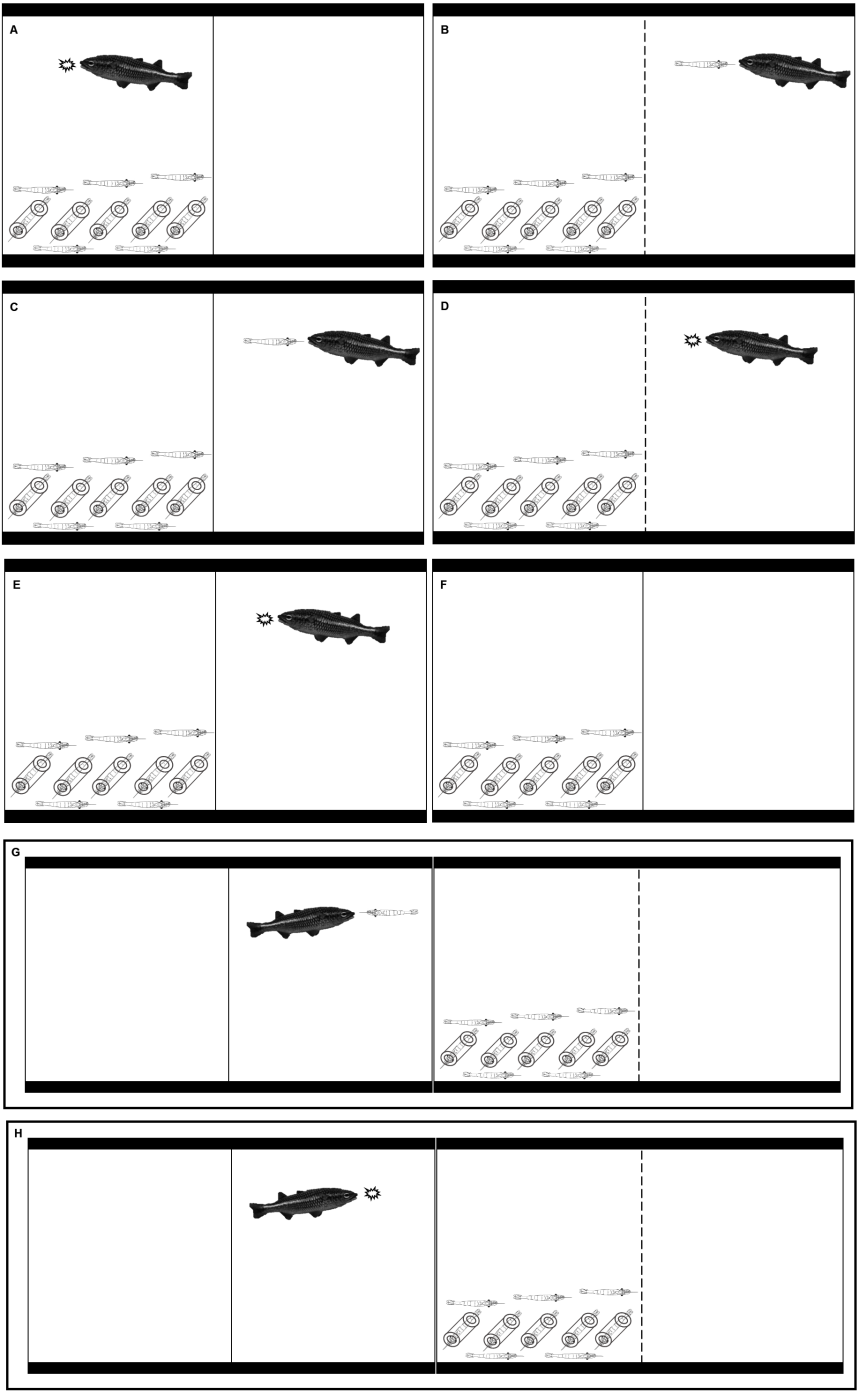
Illustrations that represent the eight treatments in our experiment. (A) KVM = kairomones + visual + mechanosensory cues; (B) KAV = kairomones + alarm + visual cues; (C) KA= kairomones + alarm cues; (D) KV: = kairomones + visual cues; (E) K = kairomone cues; (F) C = Control (no fish cues); (G) VP = visual cues from fish that ate shrimp; (H) VF = visual cues from fish that ate flakes.

Given that *A. monticola* are found in small groups in the stream reaches where they were collected (ME Ocasio-Torres, pers. obs., 2009; Kenny, 1995), we wet-transferred them in groups of 3 or 4 to 75.7-L holding aquaria. Each holding aquarium had fish that were housed for 2 weeks to give them sufficient acclimation period to laboratory conditions. The shrimp (10-20 individuals) were also acclimated in holding aquaria (also 75.7-L) two to ten days before the experiments. The fish and shrimp in the holding aquaria were fed 1 g of flake food (Omega™ One Freshwater Flakes Fish Food, OmegaSea, Bacon Road Painesville, Ohio) every two days. The holding and experimental aquaria had gravel (9 kg of CaribSea Instant Aquarium Gravel; CaribSea, Fort Pierce, Florida) to provide surface area to denitrifying bacteria. The holding and experimental aquaria were filled with filtered water up to 1 inch from the top of the aquaria. The aquaria water was kept at 23 °C. The aquaria had filters without activated carbon (API SuperClean Power Filter, Size 30, Mars Fishcare Inc., Chalfont, Pennsylvania) and a power head (Aqua Clear 20 Power Head; Rolf C Hagen Corp., Mansfield, Massachusetts) to oxygenate and circulate the water. Five PVC tubes (125 mm long, 25 mm in diameter) were added to the experimental aquaria to provide refuge space for the shrimp (Fig. 1). The aquaria had either white or clear dividers that split the tanks in half and allowed water to flow between the two compartments but prevented movement of organisms between tank halves (holes were <0.1 mm diameter) (Fig. 1). Clear dividers permitted visual and chemical exposure between tank halves, while white dividers limited visual exposure between the compartments but allowed chemical exposure (see Ocasio-Torres, Crowl & Sabat, 2014). Ten *X. elongata* were added to each experimental aquarium 24 h before the experiments started. Different shrimp were used for the different treatments and the different replicates; therefore, each individual shrimp was subject to only one replicate of one treatment. Shrimp density was higher than densities documented in natural pools; there were 10 *X. elongata* 0.18 m^−2^, which equals 55.6 *X. elongata* m^−2^. Previous studies and observations have reported *X. elongata* densities for natural streams at the Luquillo Experimental Forest that have ranged between 0 and 28.6 *X. elongata* m^−2^ (Crowl et al., 2001; Covich et al., 2009). Our experimental density was higher than the natural one in order to compensate for the reduction in experimental shrimp due to the occurrence of predation events in the KVM (kairomones + visual + mechanosensory). This treatment (see Fig. 1) had fish in the same compartment as experimental shrimp and, thus, predation of these shrimp was possible. With ten shrimp we increased the possibility of having live shrimp for the KVM treatment throughout the duration of the trials.

Eight treatments were performed to determine if the refuge use and foraging patterns of LR *X. elongata* were affected by individual cues from *A. monticola* or by multiple cues from *A. monticola* and from other *X. elongata* shrimp in danger (Table 1 and Fig. 1). Experiments started at sunset (between 1700 and 1800 depending on the time of the year). At sunset, one fish was placed in the same compartment where the shrimp were previously placed in the KVM treatment (Table 1 and Fig. 1). Predation of experimental shrimp occurred in all KVM replicates; therefore, alarm cues were present in this treatment (see Results). At sunset, one fish was also placed separate from the shrimp, in the empty compartment of the aquaria in the KAV (kairomones + alarm + visual), KA (kairomones + alarm), KV (kairomones + visual), and K (kairomones) treatments (Table 1 and Fig. 1). One fish was also placed in an adjacent aquarium that allowed visual cues but prevented chemical or mechanosensory cues for the VP (visual cues from fish that ate shrimp) and VF (visual cues from fish that ate flakes) treatments (Table 1 and Fig. 1). Finally, filtered water was transferred to the empty side of the tank in the C (control) treatment to simulate water movement from the fish addition that occurred in all other treatments (Table 1 and Fig. 1). Shrimp from all the treatments were fed with 1 g of flake food half an hour after the experiments started. At this time, the fish from the KVM, KV, K, and VF treatments were also fed with 1 g of flake food, and the fish from the KAV, KA, and VP treatments were fed with one live *X. elongata* that weighed approximately 1 g (Table 1 and Fig. 1).

**Table 1:** Description of the eight treatments in our study in relation to the type of cues, the food that was fed to the fish, the divider color, the type of barrier between fish and shrimp, and the location of fish in relation to shrimp.

**Treatment acronyms**	**Type of cues**	**Fish food**	**Divider color**	**Barrier between fish and shrimp**	**Fish location in relation to shrimp**
KVM	Kairomones + Visual + Mechanosensory	Flakes^*^	White	None	Same compartment
KAV	Kairomones + Alarm + Visual	Shrimp	Clear	Divider	Adjacent compartment of the same tank
KA	Kairomones + Alarm	Shrimp	White	Divider	Adjacent compartment of the same tank
KV	Kairomones + Visual	Flakes	Clear	Divider	Adjacent compartment of the same tank
K	Kairomones	Flakes	White	Divider	Adjacent compartment of the same tank
VP	Visual	Shrimp	Clear	Aquarium wall	Adjacent aquarium
VF	Visual	Flakes	Clear	Aquarium wall	Adjacent aquarium
C	Control (no fish or alarm cues)	Not applicable	White	Divider	No fish

**Notes.**

* Although fish were fed flakes in the KVM treatment, all fish in this treatment were able 4 to feed on experimental shrimp; therefore, alarm cues were also present in this treatment.

Discrete observations occurred one hour after sunset (between 1800 and 1900 depending on the time of the year) and at nighttime (2300) of Day 1, and at sunrise (between 0600 and 0700), and at sunset (between 1700 and 1800) of Day 2 of the experiments. These four observation times were chosen to have them correspond approximately to (1) the first moments of darkness at night, (2) the time when shrimp are very active (Pringle et al., 1993; Johnson & Covich, 2000; March et al., 1998; Scatena & Johnson, 2001; Hein, 2009; Ocasio-Torres, Crowl & Sabat, 2015a), (3) the first moments of sunlight in the morning, and (4) 24 h after fish addition, respectively. For night observations, low-intensity red lights that were 15 cm in front of each aquarium were used to observe the shrimp. Observations consisted of counting the number of shrimp in refuge, the number of shrimp that were foraging out of refuge, and the number of shrimp that were inactive out of refuge. We considered that shrimp were foraging (out of refuge) when they were actively searching for food or eating (bringing food from their chelae to their mouths) and we considered that shrimp were inactive (out of refuge) when they were standing still (neither searching for food or eating). Experiments were run in eight cycles, with each cycle consisting of one replicate for each of the eight treatments. The experiments took place between August 2015 and September 2016. Aquaria were thoroughly cleaned between experimental sets. All fish and surviving shrimp were released to their natural habitats when the experiments were finished. The Institutional Animal Care and Use Committee at Florida International University (IACUC-15-018) and at the University of Puerto Rico (3000-07-06-2015) approved all procedures.

### Data analysis

We used a generalized linear mixed-effects model (GLMMs) to examine the effects of the observation times, the treatments, and the interactions between the observation times and the treatments on the number of shrimp in refuge and on the number of shrimp that were foraging. The fixed factors of the GLMM model were the observation times, the cue treatments, and the interactions between the observation times and the cue treatments. The total number of live shrimp in the tank was treated as a covariate, and the replicates as a random block effect. We assumed a binomial error distribution and logit-link function. The GLMMs were run in the lme4 package in Infostat, which links to the R environment (R for Windows 4.0.2). We also ran Fisher’s LSD (Least Significant Difference) tests to identify means that differed when the treatments were significantly different (*P* <0.05).

## Results

### Number of live shrimp in the KVM treatment

Fish predated on experimental shrimp in all replicates from the KVM (kairomones + visual + mechanosensory) treatment (Table 2).

**Table 2:** Number of live shrimp in the KVM (kairomones + visual + mechanosensory cues) treatment in relation to the replicate number and the observation times.

**Replicate**	**Observation time**	**Live shrimp**
1	One hour after sunset of Day 1	9
	Nighttime of Day 1	8
	Sunrise of Day 2	7
	Sunset of Day 2	7
2	One hour after sunset of Day 1	10
	Nighttime of Day 1	10
	Sunrise of Day 2	10
	Sunset of Day 2	8
3	One hour after sunset of Day 1	9
	Nighttime of Day 1	9
	Sunrise of Day 2	9
	Sunset of Day 2	6
4	One hour after sunset of Day 1	10
	Nighttime of Day 1	10
	Sunrise of Day 2	9
	Sunset of Day 2	9
5	One hour after sunset of Day 1	9
	Nighttime of Day 1	9
	Sunrise of Day 2	9
	Sunset of Day 2	5
6	One hour after sunset of Day 1	10
	Nighttime of Day 1	8
	Sunrise of Day 2	8
	Sunset of Day 2	8
7	One hour after sunset of Day 1	8
	Nighttime of Day 1	8
	Sunrise of Day 2	8
	Sunset of Day 2	7
8	One hour after sunset of Day 1	8
	Nighttime of Day 1	6
	Sunrise of Day 2	6
	Sunset of Day 2	6

### Number of shrimp in refuge

**Figure 2:**
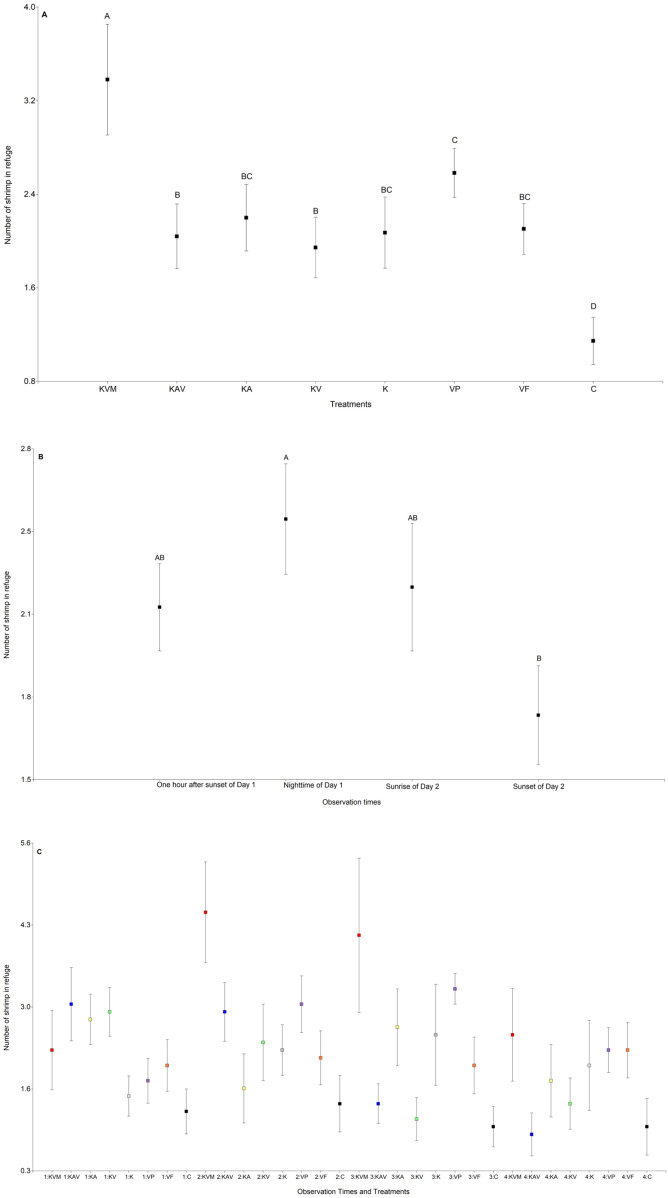
Mean (±0.95*SE) number of shrimp in refuge in relation to the treatments (A), the observation times (B), and the interaction between the treatments and the observation times (C). *X*-axis for (A) and (C) KVM = kairomones + visual + mechanosensory cues; KAV = kairomones + alarm + visual cues; KA= kairomones + alarm cues; KV = kairomones + visual cues; K = kairomone cues; VP = visual cues from fish that ate shrimp; VF = visual cues from fish that ate flakes; C = Control (no fish cues). *X*-axis for (C) 1: One hour after sunset of Day 1; 2: Nighttime of Day 1; 3: Sunrise of Day 2; 4: Sunset of Day 2. Data points with different uppercase letters are significantly different.

The treatments significantly influenced the number of shrimp in refuge (Table 3, Fig. 2A and Table S1). The number of shrimp in refuge was significantly higher in the KVM (kairomones + visual + mechanosensory) treatment than in all other treatments (Fig. 2A and Table S2). Refuge use was similar in the KAV (kairomones + alarm + visual), KA (kairomones + visual), KV (kairomones + visual), K (kairomones), and VF (visual cues from fish that ate flakes) treatments (Fig. 2A and Table S2). The number of shrimp in refuge in the C (control) treatment was lower than in all other treatments (Fig. 2A and Table S2). Observation times significantly influenced the number of shrimp in refuge; refuge use was higher at nighttime of Day 1 than at sunset of Day 2 (Table 3, Fig. 2B and Table S2). The interaction between the treatments and the observation times was also significant (Fig. 2C and Table 1). Shrimp exposed to all fish cues (KVM) hid in refuge more than shrimp in the K, VP, VF, and C treatments at nighttime, and more than shrimp in the KAV (kairomones + alarm + visual), KV (kairomones + visual) and C treatments at sunrise (Table 3, Fig. 2B and Table S3).

**Table 3:** Statistical results of the generalized linear mixed-effects model (GLMMs) with binomial distribution errors performed in our study.

**Test**	**Dependentvariable**	**Effects**	**df**	***F***	***P***
Effect of cues on the	Number of shrimp	Treatment	7	10.05	<0.0001
Number of shrimp	in refuge	Time	3	3.06	0.0292
in refuge		Time × Treatment	21	2.15	0.0033
		Error	224		
Effect of fish cues on the	Number of shrimp	Treatment	7	24.60	<0.0001
Number of shrimp that	that were foraging	Time	3	13.44	<0.0001
were foraging		Time × Treatment	21	3.37	<0.0001
		Error	224		

### Number of shrimp that were foraging out of refuge

**Figure 3:**
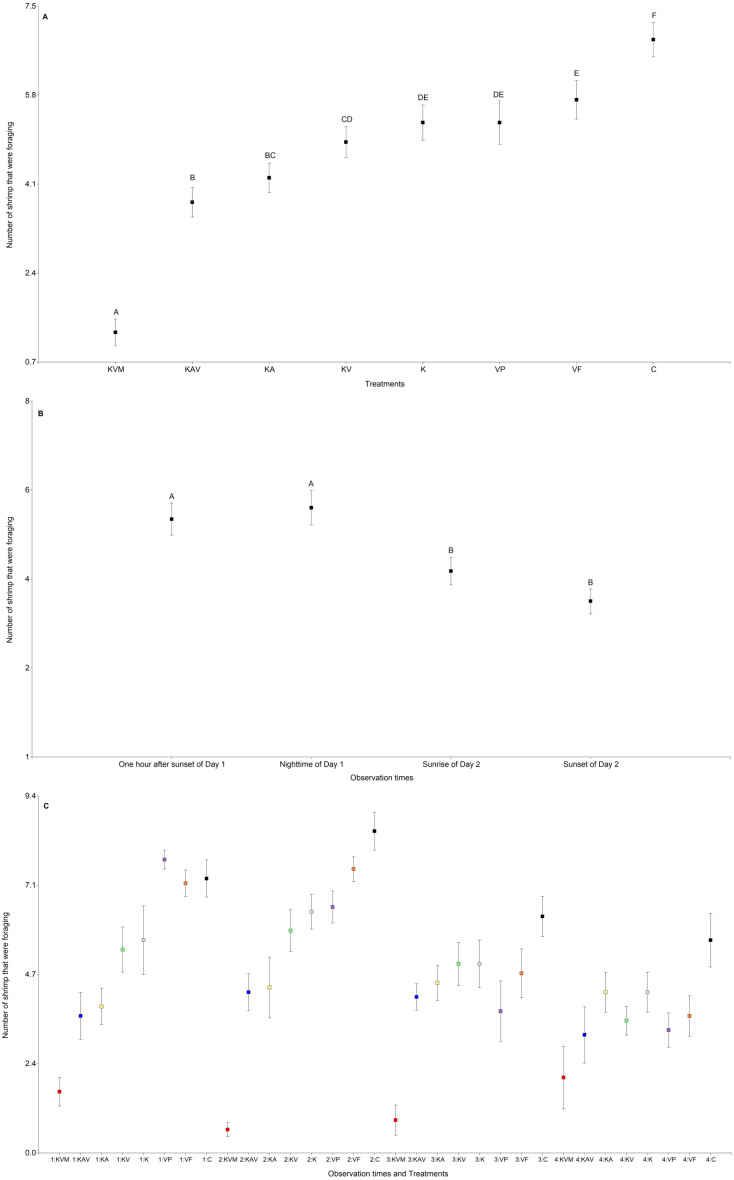
Mean (±0.95*SE) number of shrimp that were foraging in relation to the treatments (A), the observation times (B), and the interaction between the treatments and the observation times (C). *X*-axis for (A) and (C) KVM = kairomones + visual + mechanosensory cues; KAV = kairomones + alarm + visual cues; KA= kairomones + alarm cues; KV = kairomones + visual cues; K = kairomone cues; VP = visual cues from fish that ate shrimp; VF = visual cues from fish that ate flakes; C = Control (no fish cues). *X*-axis for (C) 1: One hour after sunset of Day 1; 2: Nighttime of Day 1; 3: Sunrise of Day 2; 4: Sunset of Day 2. Data points with the different uppercase letters are significantly different.

The treatments, the observation times, and the interaction between the treatments and the observation times significantly influenced the number of shrimp that were foraging (Table 3 and Fig. 3). There was an increasing trend in the number of shrimp foraging as the number of cues decreased (Fig. 3A and Table S4). There were less shrimp foraging in the KVM treatment than in all other treatments (Fig. 3A and Table S4). There were also less shrimp foraging in the KAV treatment than in the KV, K, VP, VF, and C treatments (Fig. 3A and Table S4). There were less shrimp foraging in the KA treatment than in the K, VP, VF, and C treatments (Fig. 3A and Table S4). There were also less shrimp foraging in the KV than in the VF and C treatments. Shrimp in the K, VP, and VF foraged similarly (Fig. 3A and Table S4). Shrimp in the C treatment foraged less than shrimp from all other treatments (Fig. 3A and Table S4). Regardless of the treatments, shrimp foraged similarly one hour after sunset and at nighttime but decreased their foraging at sunrise (Fig. 3B and Table S5). Shrimp in the KVM treatment foraged less than shrimp in other treatments one hour after sunset, at nighttime, and at sunrise, but foraged similarly to shrimp in the KAV, KA, KV, K, VP, and VF treatments 24 h after fish addition (Fig. 3C and Table S6).

## Discussion

Shrimp exposed to only visual (VP and VF) cues or kairomones (K) did not differ in refuge use or foraging from control (C) shrimp that were not presented any fish or alarm cue. These results were not expected and suggest that fish visual and chemical cues when presented alone are not perceived as predation threats to *X. elongata*. Another unexpected result was that shrimp exposed only to kairomones used refuge similarly to shrimp only exposed to visual cues (VP and VF). This result is contrary to our prediction, which was based on previous studies suggesting that in localities or time periods with poor visibility (such as in turbid streams or at night), chemical cues should be more useful or reliable than visual ones (Petranka, Kats & Sih, 1987; Crowl & Covich, 1990; Stauffer & Semlitsch, 1993; Dodson et al., 1994; Bouwma & Hazlett, 2001; Hickman, Stone & Mathis, 2004). An explanation for the lack of difference in refuge use between visual and kairomones cues from fish when presented singly is that they are redundant to *X. elongata* (i.e., they carry the same information or certainty about predation threat) (Partan & Marler, 2005; Weissburg, Smee & Ferner, 2014).

The result that shrimp in the KVM treatment significantly used refuge in a higher number and foraged in a lower number than shrimp in all other treatments supports our prediction that multiple cues would elicit an additive or enhancement response. Bear in mind that in all KVM replicates fish fed on experimental shrimp, thus shrimp in this treatment were, in effect, exposed to four cues: kairomones + alarm cues + visual cues + mechanosensory. Another interesting conclusion that can be drawn from the responses of shrimp to the multiple cue treatments is that there appears to be a threshold or hierarchical response in the number of cues needed to elicit a clear antipredator response in terms of refuge use. Treatments with two (KV and KA) or three (KAV) cues tended to not differ from treatments with one cue or among themselves in the number of shrimp in refuge. It appears that in *X. elongata* (at least under laboratory conditions) at least four predator cues perceived simultaneously (kairomones + alarm cues + visual cues + mechanosensory) are necessary to elicit refuge use. The effect of multiple cues on the number of shrimp foraging was different to that observed on refuge use. A significant trend towards a reduction in the number of shrimp foraging with increasing number of cues, particularly in treatments that included kairomone exposure in combination with alarm and visual cues (KAV, KA and KV), can be observed (Fig. 3 and Table S3). This difference suggests that “freezing” and stopping foraging is a more sensitive response to predator cues than refuge use. It is also probably the first response of these shrimp to the threat of predation and suggests a gradual response to predation threat similar to what has been documented in the crab *Heterozius rotundifrons* (Hazlett & Mclay, 2005). *Xiphocaris elongata* are translucent shrimp (Johnson & Covich, 2000), so reducing their mobility should reduce the likelihood of being detected by a visual predator. We envision that a foraging shrimp that starts receiving predator cues would initially freeze and cease foraging. If the cues disappear, it will continue foraging. However, only if signals indicate the almost certain presence of a predator will it seek a refuge and hide. Freezing when perceiving a moderate level of threat and subsequently resuming foraging when the threat decreases may be more energetically cost effective than stopping foraging, seeking a refuge and remaining in hiding for a length of time.

Our results add to the growing literature on how prey that detect more than one predator cue produce a greater and more complex behavioral antipredator response than prey that only detect one cue (Weissburg, Smee & Ferner, 2014). Prey that perceive predators by way of multiple sensory channels cannot only improve detection, but can estimate distance to a predator because visual, chemical, and mechanosensory signals travel at different speeds (Weissburg, Smee & Ferner, 2014). Another advantage of multiple-channel cues is reduction of habituation, or increase in certainty (Weissburg, Smee & Ferner, 2014). Habituation can happen to a *Xiphocaris elongata* shrimp continuously receiving chemical signals from upstream predators and becoming unresponsive to such cues. Overreacting to such chronic signals would not be advantageous neither if such a shrimp stops foraging and seek refuge when only smelling a predatory fish. Given persistence of such a signal, a prey behaving in such a way will most likely not grow or reproduce and might even starve to death. Alternatively, it will be adaptive to such a shrimp to stop foraging and seek refuge when simultaneously smelling, seeing, and sensing the pressure waves of a moving fish. Such a combination of signals increases certainty, unambiguously communicating imminent danger.

Finally, we accept our prediction that shrimp behavior would be affected by the observation times. *Xiphocaris elongata* shrimp are documented to be mostly nocturnal although they can be seen actively eating throughout the day (Johnson & Covich, 2000; Kikkert, Crowl & Covich, 2009; Ocasio-Torres, Crowl & Sabat, 2015a), and our results support these mostly nocturnal habits; more shrimp foraged in dark hours (1800–1900 and 2300) than in lighter hours (0600-0700 and 1700–1800). Our results also suggest an interaction between cue treatments and time periods. At nighttime (23:00), shrimp exposed to all fish cues (KVM) hid in refuge more than shrimp in the K, VP, and VF treatments. This suggests that visual cues alone lose relevance as a signal of danger at night, which makes sense. At sunrise, shrimp in the KAV, KV, and C treatments were the ones that used refuge less than the KVM shrimp. This suggests that during the day sensing a fish mechanically in combination with seeing and smelling it communicates danger more strongly than just smelling and seeing it. Thus, during the day the mechanosensory channel gains relevance. Another interesting result is that regardless of cue treatment, shrimp tended to forage less at sunrise. Visual predators such as *A. monticola* that reduce foraging at night should have high hunger and activity levels at sunrise, which makes this time-period particularly dangerous for *X. elongata.*

## Conclusions

Based on results from this and previous studies, we conclude that the ability of *Xiphocaris elongata* shrimp to detect predator signals using different sensory channels increases their defensive responses, which ultimately increases their survival (Ocasio-Torres, Crowl & Sabat, 2014; Ocasio-Torres, Crowl & Sabat, 2015a; Ocasio-Torres et al., 2015b). This study also suggests that predator detection by *X. elongata* can, indirectly, alter the effects of predatory fishes on the energy flow of the stream ecosystem, given that decreased foraging and increased hiding time may reduce the amount of leaf-litter shredded by these shrimp (Ocasio-Torres, Crowl & Sabat, 2015a). Studies that examine the effect of other predators (e.g., other fishes, the decapod *Macrobrachium* spp.) on amphidromous shrimp like *X. elongata* and on *Atya* spp. (shrimp from the Atyidae family) are recommended to understand a more complete spectrum of predator–prey dynamics and their consequences at the community and ecosystem level in Caribbean streams. Understanding how aquatic prey detect and generate a behavioral response to their potential predators is essential for comprehending how animal interactions impact aquatic communities and ecosystems and crucial for the general understanding of the ecological and evolutionary mechanisms that regulate predator–prey interactions.

##  Supplemental Information

10.7717/peerj.11011/supp-1 Supplemental Information 1 Fisher’s LSD (Least Significant Difference) test results for the effect of the treatments on the number of shrimp in refuge KVM = kairomones + visual + mechanosensory cues; KAV = kairomones + alarm + visual cues; KA= kairomones + alarm cues; KV = kairomones + visual cues; K = kairomone cues; VP = visual cues from fish that ate shrimp; VF = visual cues from fish that ate flakes; C = Control (no fish cues).

10.7717/peerj.11011/supp-2 Supplemental Information 2 Fisher’s LSD (Least Significant Difference) test results for the effect of the observation times on the number of shrimp in refuge

10.7717/peerj.11011/supp-3 Supplemental Information 3 Fisher’s LSD (Least Significant Difference) test results for the effect of the interaction between the treatments and the observation times on the number of shrimp in refuge KVM = kairomones + visual + mechanosensory cues; KAV = kairomones + alarm + visual cues; KA= kairomones + alarm cues; KV = kairomones + visual cues; K = kairomone cues; VP = visual cues from fish that ate shrimp; VF = visual cues from fish that ate flakes; C = Control (no fish cues)

10.7717/peerj.11011/supp-4 Supplemental Information 4 Fisher’s LSD (Least Significant Difference) test results for the effect of the treatments on the number of shrimp that were foraging KVM = kairomones + visual + mechanosensory cues; KAV = kairomones + alarm + visual cues; KA= kairomones + alarm cues; KV = kairomones + visual cues; K = kairomone cues; VP = visual cues from fish that ate shrimp; VF = visual cues from fish that ate flakes; C = Control (no fish cues)

10.7717/peerj.11011/supp-5 Supplemental Information 5 Fisher’s LSD (Least Significant Difference) test results for the effect of the observation times on the number of shrimp that were foraging

10.7717/peerj.11011/supp-6 Supplemental Information 6 Fisher’s LSD (Least Significant Difference) test results for the effect of the interaction between the treatments and the observation times on the number of shrimp that were foraging KVM = kairomones + visual + mechanosensory cues; KAV = kairomones + alarm + visual cues; KA = kairomones + alarm cues; KV = kairomones + visual cues; K = kairomone cues; VP = visual cues from fish that ate shrimp; VF = visual cues from fish that ate flakes; C = Control (no fish cues)

10.7717/peerj.11011/supp-7 Supplemental Information 7 Raw data

10.7717/peerj.11011/supp-8 Supplemental Information 8 Results for the GLMM models and Fisher’s LSD tests in R languange

## Additional Information and Declarations

### Field Study Permissions

The following information was supplied relating to field study approvals (i.e., approving body and any reference numbers): The animal collection methods in this research were approved by the Department of Natural and Environmental Resources of Puerto Rico with the permit numbers O-VS-PVS15-SJ-00728-10022015 and R-VS-PVS15-SJ-00516-11042016.

### Data Availability

The following information was supplied regarding data availability: Raw data are available in the Supplemental Files.
