# Supplementary material for: ­­Effect of multimodal cues from a predatory fish on refuge use and foraging on an amphidromous shrimp
Source: PeerJ. 2021 Mar 12;9:e11011. doi: 10.7717/peerj.11011 (PMC7958891; doi:10.7717/peerj.11011)

**Refuge**

C:\Users\maria\OneDrive - Universidad Ana G. Mendez\A Post-doc\Exp crevice use\Peer-J\Más reciente\Datos crudos_MOcasio.IDB2 : 10/12/2020 - 9:52:41 PM - [Version : 4/30/2020] - [R 4.0.2]

**Generalized linear mixed effects models**

**R specification of the model**

*glmm.model.000_Refuge_ML<-glmer(cbind(Refuge*

*,as.numeric(as.character(Shrimp))-Refuge)~1+Treatment+Time+Treatment:Time+(1|Replicate)*

*,family=myFamily*

*,na.action=na.omit*

*,nAGQ=1*

*,data=R.data00)*

**Results for model: glmm.model.000_Refuge_ML**

*Dependent variable: Refuge*

**General**

Family Link nAGQ

binomial logit 1

**Fit measurements**

N AIC BIC logLik Deviance

256 937.35 1054.34 -435.68 356.82

*Smaller AIC and BIC is better*

**Wald test for fixed effects**

Source numDF denDF F-value p-value

Treatment 7 224 10.05 <0.0001

Time 3 224 3.06 0.0292

Treatment:Time 21 224 2.15 0.0033

**Random effects parameters**

RndEff Param Var SD

Replicate (Intercept) 0.02 0.15

**Refuge - Adjusted means and standard error for the levels of Treatment**

*Inverse link function with random effects=0*

*LSD Fisher (Alpha:=0.05)*

*p-value correction procedure: No*

Treatment LinPred S.E. Mean S.E.

KVM -0.41 0.14 0.40 0.03 A

VP -1.09 0.14 0.25 0.03 B

KA -1.30 0.15 0.21 0.02 B C

VF -1.34 0.15 0.21 0.02 B C

K -1.37 0.15 0.20 0.02 B C

KAV -1.49 0.16 0.18 0.02 C

KV -1.50 0.16 0.18 0.02 C

C -2.05 0.18 0.11 0.02 D

*Means with a common letter are not significantly different (p > 0.05)*

**Refuge - Adjusted means and standard error for the levels of Time**

*Inverse link function with random effects=0*

*LSD Fisher (Alpha:=0.05)*

*p-value correction procedure: No*

Time LinPred S.E. Mean S.E.

2 -1.11 0.11 0.25 0.02 A

3 -1.30 0.12 0.21 0.02 A B

1 -1.31 0.11 0.21 0.02 A B

4 -1.56 0.12 0.17 0.02 B

*Means with a common letter are not significantly different (p > 0.05)*

**Refuge - Adjusted means and standard error for the levels of Treatment*Time**

*Inverse link function with random effects=0*

*LSD Fisher (Alpha:=0.05)*

*p-value correction procedure: No*

Treatment Time LinPred S.E. Mean S.E.

KVM 2 0.11 0.25 0.53 0.06 A

KVM 3 -0.01 0.25 0.50 0.06 A

KVM 4 -0.60 0.28 0.35 0.07 A B

VP 3 -0.73 0.25 0.32 0.05 B C

VP 2 -0.85 0.25 0.30 0.05 B C D

KAV 1 -0.85 0.25 0.30 0.05 B C D

KAV 2 -0.91 0.25 0.29 0.05 B C D E

KV 1 -0.91 0.25 0.29 0.05 B C D E

KA 1 -0.97 0.26 0.27 0.05 B C D E F

KA 3 -1.04 0.26 0.26 0.05 B C D E F G

K 3 -1.10 0.26 0.25 0.05 B C D E F G

KVM 1 -1.13 0.28 0.24 0.05 B C D E F G H

KV 2 -1.17 0.27 0.24 0.05 B C D E F G H

VF 4 -1.24 0.27 0.22 0.05 B C D E F G H I

VP 4 -1.24 0.27 0.22 0.05 B C D E F G H I

K 2 -1.24 0.27 0.22 0.05 B C D E F G H I

VF 2 -1.32 0.28 0.21 0.05 B C D E F G H I J

VF 1 -1.39 0.29 0.20 0.05 C D E F G H I J

VF 3 -1.39 0.29 0.20 0.05 C D E F G H I J

K 4 -1.39 0.29 0.20 0.05 C D E F G H I J

VP 1 -1.52 0.30 0.18 0.04 D E F G H I J K

KA 4 -1.56 0.30 0.17 0.04 D E F G H I J K

KA 2 -1.65 0.31 0.16 0.04 E F G H I J K

K 1 -1.74 0.32 0.15 0.04 F G H I J K

KAV 3 -1.84 0.33 0.14 0.04 G H I J K

KV 4 -1.84 0.33 0.14 0.04 G H I J K

C 2 -1.84 0.33 0.14 0.04 G H I J K

C 1 -1.95 0.34 0.12 0.04 H I J K

KV 3 -2.07 0.36 0.11 0.04 I J K

C 3 -2.21 0.38 0.10 0.03 J K

C 4 -2.21 0.38 0.10 0.03 J K

KAV 4 -2.35 0.40 0.09 0.03 K

*Means with a common letter are not significantly different (p > 0.05)*


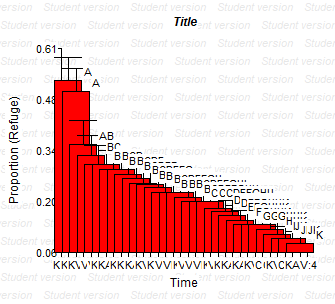


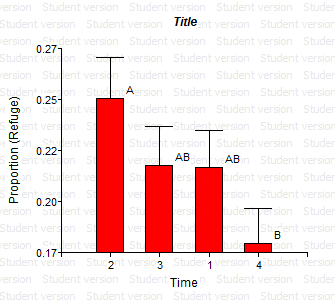


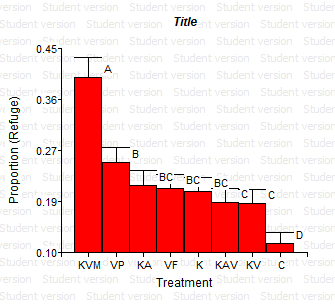


**Foraging**

C:\Users\maria\OneDrive - Universidad Ana G. Mendez\A Post-doc\Exp crevice use\Peer-J\Más reciente\Datos crudos_MOcasio.IDB2 : 10/12/2020 - 10:07:57 PM - [Version : 4/30/2020] - [R 4.0.2]

**Generalized linear mixed effects models**

**R specification of the model**

*glmm.model.001_Foraging_ML<-glmer(cbind(Foraging*

*,as.numeric(as.character(Shrimp))-Foraging)~1+Treatment+Time+Treatment:Time+(1|Replicate)*

*,family=myFamily*

*,na.action=na.omit*

*,nAGQ=1*

*,data=R.data01)*

**Results for model: glmm.model.001_Foraging_ML**

*Dependent variable: Foraging*

**General**

Family Link nAGQ

binomial logit 1

**Fit measurements**

N AIC BIC logLik Deviance

256 974.07 1091.06 -454.03

*Smaller AIC and BIC is better*

**Wald test for fixed effects**

Source numDF denDF F-value p-value

Treatment 7 224 24.60 <0.0001

Time 3 224 13.44 <0.0001

Treatment:Time 21 224 3.37 <0.0001

**Random effects parameters**

RndEff Param Var SD

Replicate (Intercept) 0.00 0.00

**Foraging - Adjusted means and standard error for the levels of Treatment**

*Inverse link function with random effects=0*

*LSD Fisher (Alpha:=0.05)*

*p-value correction procedure: No*

Treatment LinPred S.E. Mean S.E.

C 0.87 0.13 0.70 0.03 A

VF 0.34 0.12 0.58 0.03 B

VP 0.15 0.12 0.54 0.03 B C

K 0.13 0.11 0.53 0.03 B C

KV -0.03 0.11 0.49 0.03 C D

KA -0.30 0.11 0.42 0.03 D E

KAV -0.50 0.12 0.38 0.03 E

KVM -1.78 0.19 0.14 0.02 F

*Means with a common letter are not significantly different (p > 0.05)*

**Foraging - Adjusted means and standard error for the levels of Time**

*Inverse link function with random effects=0*

*LSD Fisher (Alpha:=0.05)*

*p-value correction procedure: No*

Time LinPred S.E. Mean S.E.

2 0.16 0.10 0.54 0.02 A

1 0.12 0.09 0.53 0.02 A

3 -0.35 0.09 0.41 0.02 B

4 -0.50 0.09 0.38 0.02 B

*Means with a common letter are not significantly different (p > 0.05)*

**Foraging - Adjusted means and standard error for the levels of Treatment*Time**

*Inverse link function with random effects=0*

*LSD Fisher (Alpha:=0.05)*

*p-value correction procedure: No*

Treatment Time LinPred S.E. Mean S.E.

C 2 1.73 0.31 0.85 0.04 A

VP 1 1.24 0.27 0.77 0.05 A B

VF 2 1.10 0.26 0.75 0.05 A B C

C 1 0.97 0.25 0.72 0.05 A B C D

VF 1 0.91 0.25 0.71 0.05 B C D E

VP 2 0.62 0.23 0.65 0.05 B C D E F

K 2 0.56 0.23 0.64 0.05 B C D E F

C 3 0.51 0.23 0.62 0.05 C D E F G

KV 2 0.35 0.23 0.59 0.06 D E F G H

K 1 0.25 0.23 0.56 0.06 E F G H I

C 4 0.25 0.23 0.56 0.06 E F G H I

KV 1 0.15 0.22 0.54 0.06 F G H I J

K 3 0.00 0.22 0.50 0.06 F G H I J K

KV 3 0.00 0.22 0.50 0.06 F G H I J K

VF 3 -0.10 0.22 0.48 0.06 G H I J K L

KA 3 -0.20 0.22 0.45 0.06 H I J K L M

KA 2 -0.25 0.23 0.44 0.06 H I J K L M

KAV 2 -0.30 0.23 0.43 0.06 I J K L M

K 4 -0.30 0.23 0.43 0.06 I J K L M

KA 4 -0.30 0.23 0.43 0.06 I J K L M

KAV 3 -0.35 0.23 0.41 0.06 I J K L M

KA 1 -0.46 0.23 0.39 0.05 J K L M

VP 3 -0.51 0.23 0.38 0.05 K L M

KAV 1 -0.56 0.23 0.36 0.05 K L M

VF 4 -0.56 0.23 0.36 0.05 K L M

KV 4 -0.62 0.23 0.35 0.05 K L M

VP 4 -0.73 0.24 0.33 0.05 L M

KAV 4 -0.79 0.24 0.31 0.05 M N

KVM 4 -0.92 0.30 0.29 0.06 M N

KVM 1 -1.53 0.31 0.18 0.04 N O

KVM 3 -2.13 0.40 0.11 0.04 O

KVM 2 -2.53 0.46 0.07 0.03 O

*Means with a common letter are not significantly different (p > 0.05)*


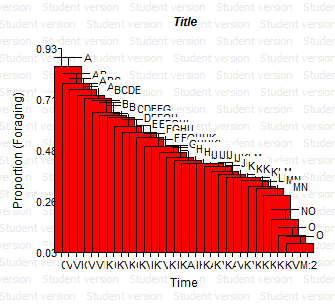


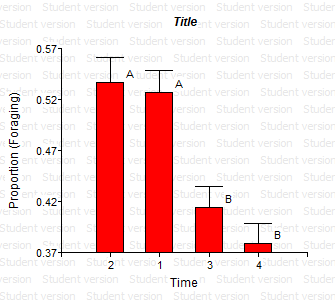


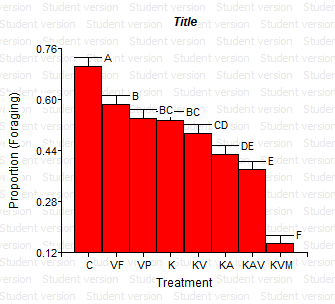

Supplement: Supplemental Information 8 [file peerj-09-11011-s008.docx]
